# Fabrication of Microfluidic Tesla Valve Employing Femtosecond Bursts

**DOI:** 10.3390/mi13081180

**Published:** 2022-07-26

**Authors:** Deividas Andriukaitis, Rokas Vargalis, Lukas Šerpytis, Tomas Drevinskas, Olga Kornyšova, Mantas Stankevičius, Kristina Bimbiraitė-Survilienė, Vilma Kaškonienė, Audrius Sigitas Maruškas, Linas Jonušauskas

**Affiliations:** 1 Femtika Ltd., Saulėtekio Ave. 15, LT-10224 Vilnius, Lithuania; devidas@femtika.lt (D.A.); rokas@femtika.lt (R.V.); linas.jon@gmail.com (L.J.); 2 Laser Research Center, Vilnius University, Saulėtekio Ave. 10, LT-10223 Vilnius, Lithuania; 3 Institute of Chemistry, Vilnius University, Naugarduko 24, LT-03225 Vilnius, Lithuania; lukas.serpytis95@gmail.com; 4 Instrumental Analysis Open Access Centre, Vytautas Magnus University, Vileikos 8, LT-44404 Kaunas, Lithuania; tomas.drevinskas@vdu.lt (T.D.); olga.kornysova@vdu.lt (O.K.); mantas.stankevicius@vdu.lt (M.S.); kristina.bimbiraite-surviliene@vdu.lt (K.B.-S.); vilma.kaskoniene@vdu.lt (V.K.)

**Keywords:** femtosecond bursts, Tesla valve, glass ablation

## Abstract

Expansion of the microfluidics field dictates the necessity to constantly improve technologies used to produce such systems. One of the approaches which are used more and more is femtosecond (fs) direct laser writing (DLW). The subtractive model of DLW allows for directly producing microfluidic channels via ablation in an extremely simple and cost-effective manner. However, channel surface roughens are always a concern when direct fs ablation is used, as it normally yields an RMS value in the range of a few µm. One solution to improve it is the usage of fs bursts. Thus, in this work, we show how fs burst mode ablation can be optimized to achieve sub-µm surface roughness in glass channel fabrication. It is done without compromising on manufacturing throughput. Furthermore, we show that a simple and cost-effective channel sealing methodology of thermal bonding can be employed. Together, it allows for production functional Tesla valves, which are tested. Demonstrated capabilities are discussed.

## 1. Introduction

Since the start of fluid manipulation in microelectromechanical systems (MEMS) [1], microfluidics systems have received substantial attention from academia. Microfluidics systems are defined in the working range of small volumes (pl-ml). These systems are revolutionary in life sciences and industry [2] thanks to low reagent consumption, high sensitivity, rapid detection, integration capability, and being highly portable [3]. Such systems found many different applications, such as synthesis of nanofiber and nanoparticles [4,5], electrochemical/biochemical sensors [6], or cell/molecular biology [7].

Nowadays, there are a lot of technologies that are capable of producing microfluidic elements. If a simple, polymer/plastic-based system is acceptable, methodologies like soft lithography [8,9], 3D printing [10,11], injection molding [2,12] or xurography [13,14] can be applied. The ultimate goal is that a prototype created in a lab has to find its application in the industry. Therefore, fabrication efficiency and cost are crucial factors. This brings some complications if glass or other hard material needs to be used, as it rules out most of the cheap alternatives. Then, one technology stands out—femtosecond (fs) laser ablation. By taking advantage of this technique, one can avoid multiple steps in manufacturing the element. For example, the selective glass etching technique requires an additional step after processing material with laser radiation. Affected material areas by laser are etched out by using dangerous acids and alkalines [15]. Ablation technology is quite simple; the laser irradiates the material and locally evaporates it. Thus far, it was shown to be capable of producing structures in plastics [16], glasses [17], and metals [18]. If the microfluidic element is not fabricated in the volume of material, it needs to be sealed, so the injected fluid would not escape the system. There are some ways to complete this stage; for example, one may use the direct thermal bonding technique [19]. In simple words, a material is heated to a melting point and applied to the sample surface, and as a result, a hermetic seal is created. Additionally, recent developments in laser technology allowed us to achieve so-called fs bursts [20]. It was shown to be a powerful addition to already highly flexible fs laser processing [21]. It can increase processing throughput [22] or the quality of the final structure [23]. As a result, there is a drive to employ this methodology in as many application fields as possible. Nevertheless, fs bursts are highly underutilized in glass microfluidic system manufacturing.

All these nuances point to the necessity of creating technology that can efficiently produce cm-scale glass microchannel systems with high quality i.e., surface roughness below a few µm. This work is dedicated to showing the capabilities of fs bursts in manufacturing glass microfluidic channels via ablation. We show how channel surface can be optimized by tuning burst parameters. As an example structure, we chose a Tesla valve. In 1920, Nikola Tesla introduced a valvar conduit—a passive valve with no moving parts. The working principle of such an element is that forward flow experiences hydraulic resistance since it is flowing through loops. On the other side, reverse flow experiences little to no hydraulic resistance. Because it has no moving parts, this design became attractive in the field of microfluidics [24]. Over the years, Tesla valves have been realized in different applications. For example, in the hydrogen fuel cell research field, Jin et al. showed the possibility to exploit the valve as a decompression unit. It was found that high-pressure hydrogen storage can increase the recharge mileage [25]. Although it is needed to decompress the hydrogen before it enters the fuel cell, the Tesla valve turned out to be a suitable element for such a process since it does not have moving parts. In addition, due to its unique geometry, when the hydrogen flows in a reverse direction, it experiences a large pressure drop between inlet and outlet. Another application is exploiting the Tesla valve in the micropump. Garcia-Morales et al. showed the possibility to construct a Tesla valve thermocavitation-based micropump [26]. We chose this structure as it is very sensitive to cut quality. Demonstration of the functionality of such valve in microscale is provided, showing that the presented burst-mode glass ablation is a viable tool to produce high-quality glass microfluidic parts.

## 2. Materials and Methods

### 2.1. Experimental Setup

**Figure 1:**
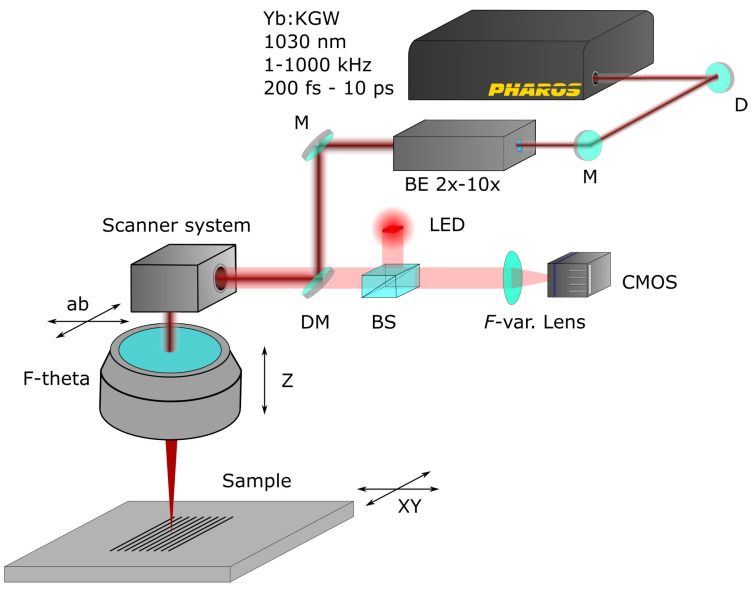
Schematic of subtractive laser workstation. M—mirror, BE—beam expander, BS—beamsplitter, F-var—variable focal distance lens.

The fabrication of the Tesla valve was completed using the “Laser Nanofactory” (Femtika) setup. It can be used for both subtractive manufacturing of microchannels and for integrating macromolecule separators. The main differences are used optics and laser parameters (tuned via software). The subtractive laser workstation setup was used in this work. It is schematically visualized in Figure 1. More information on the peculiarities of the system can be found elsewhere [27].

**Figure 2:**
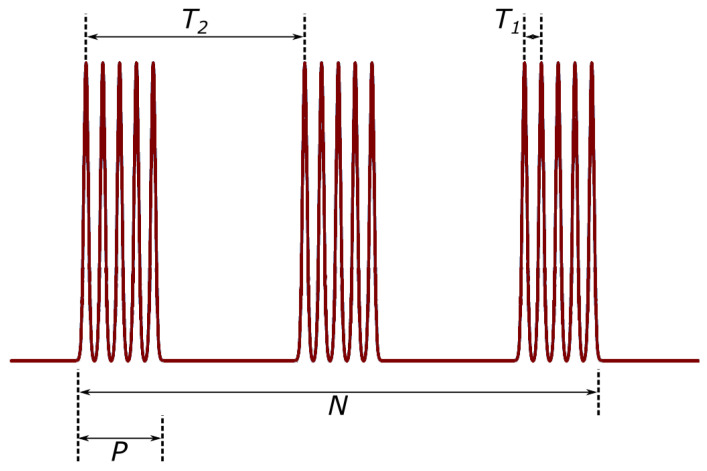
Schematic of burst principle. A single pulse is divided into a number of sub-pulses (*P*) and a number of burst packets (*N*). This whole packet, consisting of burst packets is repeated at a laser repetition rate. $T_{1}$ and $T_{2}$ indicate intra-burst time. For the laser used in the experimental setup, *P* range is from 1 to 25, *N* range— from 1 to 9. When *P* = 1 and *N* is changed, we say the laser is in an MHz burst regime. On the contrary, when *N* = 1 and *P* is changed, the laser is in the GHz burst regime. When *P* and *N* are changed, the laser is in the bi-burst regime.

One of the primary innovations tested in this work was the usage of fs bursts. They allow a controlled number of pulses and pulse packages in time. The principle of this temporal control as well as how different parameters concerning it are denoted are shown in Figure 2.

### 2.2. 3D Models

**Figure 3:**
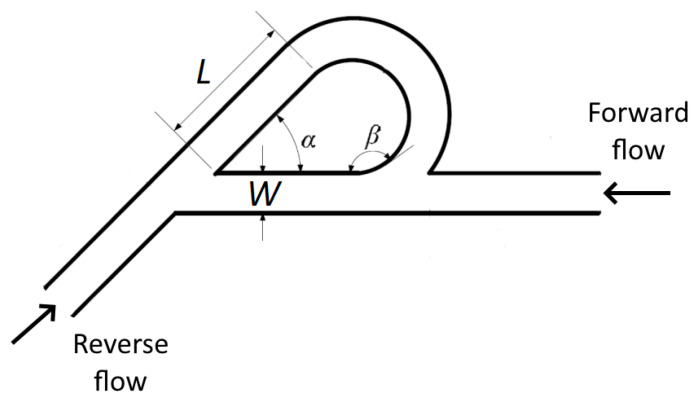
The basic scheme of Tesla valve design indicates different dimensions and parameters—here, *W*—valve width, *L*—length of the straight segment of the valve channel, α—valve side-channel leaving angle, and β—valve side-channel return angle.

**Figure 4:**
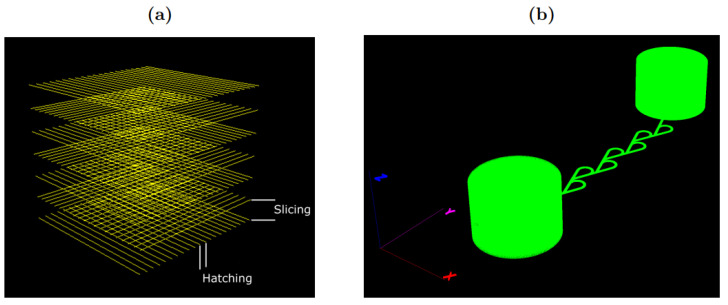
(**a**) sliced/hatched cube in 3DPoli software for a better explanation of parameters, since they are crucial for high-quality structure manufacturing; (**b**) processed Tesla valve 3D model and ready to be fabricated. Green-colored figures indicate a laser path with a shutter turned on.

To make sure that the proportions of the Tesla valve are adequate to achieve the functionality, we used one of the configurations presented in [28]. The modeling of the Tesla valve was inspired by Zhang et al., who performed a performance simulation of the element to optimize it. They have simulated valves in four different aspect ratios; one of them was realized and manufactured. Dimensions of the Tesla valve with the aspect ratio of 2 are presented in the Table 1 below, as well as the schematic model of the valve (Figure 3). This case was chosen as it allows very simple fabrication using the fs DLW setup used in this work. The model was created in Solidworks 2021 and afterward exported as STL. The created model was processed by 3DPoli software, which allows controlling all the required fabrication parameters: slicing/hatching steps (Figure 4) scanning speed, laser power, and of course the scanned geometry.

### 2.3. Sample Characterization

After fabrication, the channels were cleaned in an ultra-sound bath in order to remove any ablation debris. Then, the channel surface roughness was investigated by using an optical profilometer Bruker ContourGT-X. Each channel’s roughness was measured by scanning a 5 × 5 µm^2^ area in five random spots of the channel. Samples were also characterized using a scanning electron microscope (SEM) Hitachi SU-70 ÄKTAdesign Pump P-900 was applied to deduce the functionality of the Telsa valve.

## 3. Results and Discussion

### 3.1. Fabrication of the Microchannel System

We began our work by optimizing glass ablation parameters. The microchannel system was produced on a 1 mm thickness commercially available microscope glass slides surface (soda-lime, Thermo Scientific. Such material was chosen for production since it is chemically inert, cheap, robust, and transparent, which provides the ability to see a microfluidic system from the front and back sides. The whole device consisted of two main parts—a channel, which will act as a Tesla valve, and inlets/outlets. While channel surface roughness is important for the Tesla valve, as it influences the flow of liquid, inlets and outlets just have to be hollow. Additionally, inlets/outlets are much bigger than the channel system. Thus, they can be produced faster with little regard to wall surface roughness. Having in mind these specifications, the fabrication parameters were divided into two parts: microchannels and inlets/outlets. The microchannels were fabricated by scanning the laser beam with galvoscanners at 0.5 m/s, hatching/slicing steps of 0.3 µm and 5 µm accordingly. By using a smaller hatching step, better surface roughness was preserved. As for the inlets/outlets, the surface roughness or edge quality was not important, so the fabrication parameters were optimized fabrication time-wise. The inlets/outlets were manufactured by scanning the beam at 0.5 m/s, hatching/slicing steps of 8 µm and 4 µm, accordingly. Afterward, the fabricated microfluidic microchannel systems were washed in an ultra-sound bath to remove ablation debris that was not removed by pressurized air during the fabrication.

**Figure 5:**
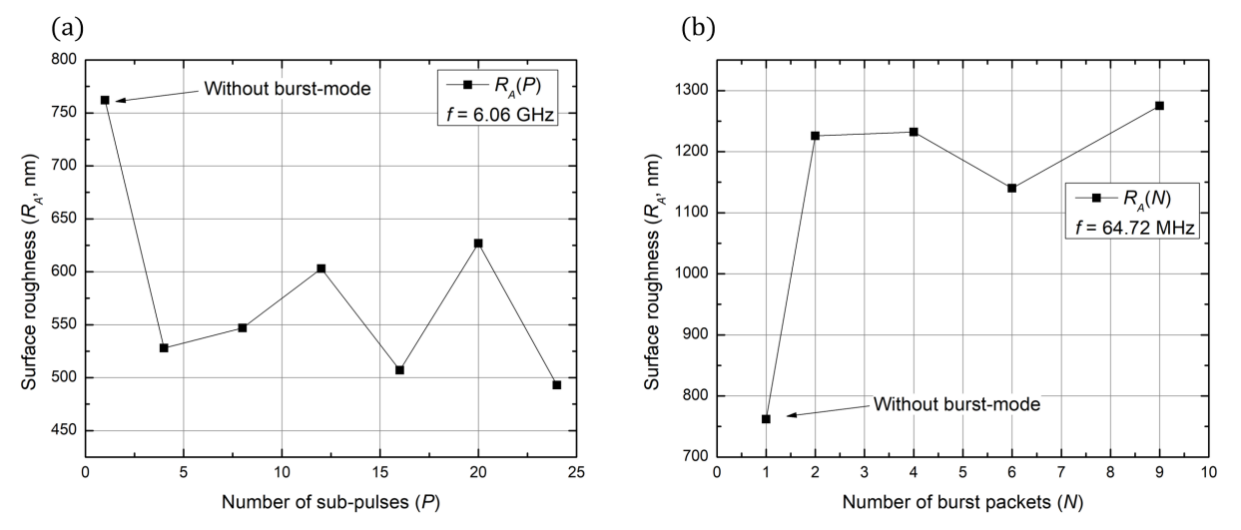
(**a**) Graphically depicted observed surface roughness values by changing sub-pulse number; (**b**) graphically depicted observed surface roughness values by changing the number of burst packets.

To have better quality ablated structure, one may try to improve the surface roughness of ablated transparent dielectric materials by inducing burst-mode ablation. The ability to generate bursts enables the possibility to divide single pulse energy into sub-pulses and create so-called burst trains. By varying burst parameters, it is possible to achieve two processing mechanisms: GHz and MHz pulse regimes. The GHz pulse regime is achieved by varying the number of burst packets (*P*), while the MHz regime is enabled by a varying number of sub-pulses (*N*). In some lasers, GHz and MHz regimes may be combined to achieve a burst-in-burst or bi-burst processing mechanism. In this work, surface roughness dependency on different processing mechanisms (GHz, MHz, and bi-burst) was investigated by ablating a square on the soda-lime glass surface (500 × 500 × 50 µm). The surface roughness was evaluated by observing arithmetical mean height (*R_A_*), which in simple terms is the average of sample surface height measured area. First, GHz processing was investigated. A parameter of *P* was varied (*N* = 1): 4, 8, 12, 16, 20, and 24. Different surface roughness values for the *P* parameter were observed and depicted graphically (Figure 5a). To understand the difference between simple ablation and burst-mode ablation, one sample without burst-mode was fabricated. From Figure 5a, we can state that GHz bursts improve the surface roughness by around 30%. The variation of surface roughness values from 500 to 625 nm can be explained by having in mind that measurements were taken in a 5 µm^2^ area chosen randomly in the sample.

Moreover, MHz processing was investigated in the same way as GHz processing. Different values of *N* were experimented: 2, 4, 6, and 9. Observed surface roughness values were depicted graphically in Figure 5b. From the data, we can see that MHz processing worsens surface roughness values by around 50%. Finally, different bi-burst configurations were tried. Various experiment configurations with resulting surface roughness values are presented in Table 2. From the results, we can see that, by exploiting the bi-burst function and using *P*2*N*2 configuration, it is possible to get surface roughness lower than 500 nm. In addition, it is important to note that, when *N* parameter is increased (MHz regime), the surface roughness increases accordingly. This dependency coincides with the graph in Figure 5b. On the other hand, we can see from Figure 5a that an increase in *P* results in better surface quality. Unfortunately, the bi-burst configuration *P*12*N*2 shows a different result. The acquired values are higher than using lower *P*. In the context of microfluidics, surface roughness plays an important role when talking about flow resistance. As literature shows, rougher surfaces introduce significant flow resistance [29]. To avoid this, scientists are using SLE, which offers the capability to produce structures with surface roughness below 200 nm [30]. The surface quality criteria are one of the key disadvantages of femtosecond laser ablation. The technology performs in higher fabrication time but lacks in preserving sub-micron surface roughness [31]. The results of this investigation open a new discussion of the possibility to ablate structures with surface roughness values below 500 nm by employing bi-burst mode processing techniques.

**Figure 6:**
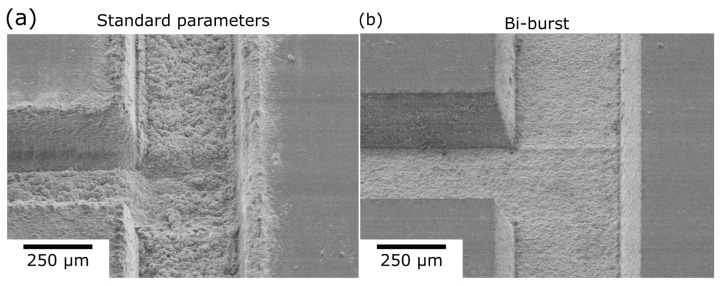
SEM images of channels produced using (**a**) standard fs laser and (**b**) bi-burst regime. Improvement of RMS from few µm to sub-500 nm is evident.

To demonstrate this difference visually, an example microfluidic channel was produced. It was manufactured either using non-burst ablation, with parameters specified in our previous works [31], or by using a newly discovered high-quality bi-burst methodology. In addition, we chose the T geometry channel to demonstrate the possibility to acquire sharp corners during processing. While it is not relevant for this work, such geometry is very popular in microfluidics, for instance for the integration of microparticle sorters [32,33]. The results are shown in Figure 6. Evidently, the bi-burst regime performed very well, with well-defined walls and corners. As a result, bi-burst was used for Tesla valve manufacturing, as smooth, uninterrupted flow is critical to avoid any channel-induced irregularities during the measurement.

**Figure 7:**
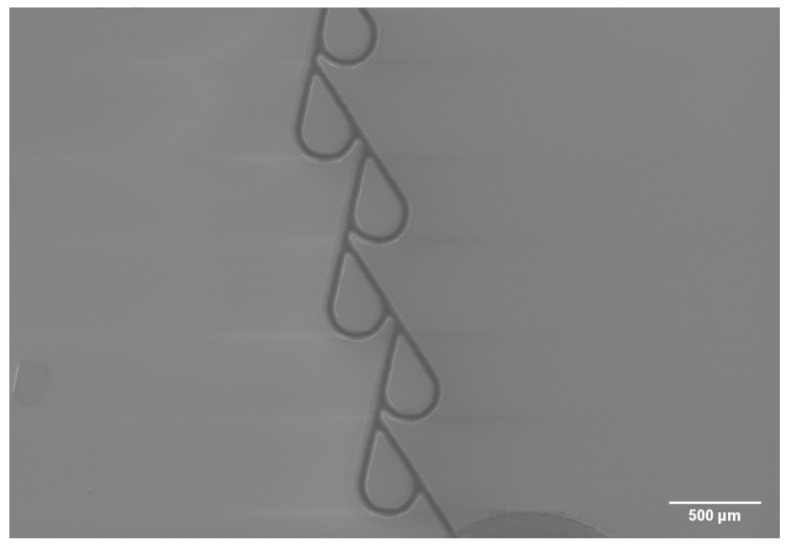
SEM image of final Tesla valve before sealing.

The production of Tesla valve followed. The SEM image of produced channel system sealing is given in Figure 7. Such a 5-stage Tesla valve was produced in 15 min (including inlets and outlets), showing that such structures can be produced relatively quickly and with quality tinkered for the specific component (high-precision valve channel, fast cutting of inlets/outlets).

### 3.2. Sealing of Tesla Valve

There are many ways to seal a micro-fluidical chip. All these methodologies differ in their implementation, required equipment, amount of technological steps, compatible materials, and bonding strength. As one of the most established methodologies for microfluidic fabrication is soft lithography [34], one may use polydimethylsiloxane (PDMS) for sealing. It is a cost-effective material that has properties to be permeable for gas, but not liquid. Such a sealing method is favorable not only due to cost but the versatility as well. The possibility to use PDMS as a sealing material is reasoned by well adhesive properties to the sample surface. In addition, PDMS is suitable to seal different materials, such as plastic, metals, or dielectrics to name a few [35]. On the other hand, the formation of bubbles and the dependence to shrink makes PDMS not such a perfect candidate for sealing microfluidic devices. Additionally, to realize it, additional equipment is needed. In addition, the process involves multiple technological steps, which might result in lower repeatability if there are even minor deviations from the bonding protocol.

Another sealing technique that is more complicated is femtosecond laser welding [15]. As the name suggests, this technique involves laser radiation with wavelengths usually in near-infrared (NIR). Laser with high repetition-rate pulses creates high-temperature pools, in which two materials can be welded together with minimal material displacement, low mechanical stress, and width of welding seam in the micrometer range. It is also a direct methodology, which does not require any additional materials in the interface or post-processing. Such technique may be used in areas where the conveyer fabrication method is realized, since, with a single laser source, one may ablate a micro-fluidic device and seal the device. It can also bond different kinds of materials like dissimilar glasses [36], or glasses with metals [37]. In addition, laser welding allows for achieving up to 95% breaking resistance [38]. In addition, it is shown to be capable of strengthening other bonding techniques [39]. Unfortunately, as mentioned before, this technique is complicated. The sample and sealing glass require having an optical surface interface which is hard to achieve due to small debris and dust [40]. As a result, bonding relatively big samples, such as cm-scale microfluidic systems, is extremely challenging.

**Figure 8:**
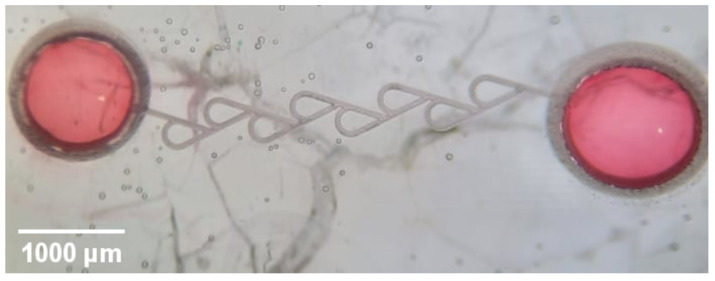
Optical microscope image of Tesla valve testing for any leaks by injecting red-colored water. No leaks or blockages can be detected, showing superb sealing through the whole cm-sized chip.

Another way is to use thermoplastics. They are highly attractive for the microfluidic system sealing process since they are cost-efficient and do not need complex preparation. In addition, they excel in good chemical properties and optical clarity. Thermoplastics have already been used in microfluidic device sealing, and results show great adhesion and hermetic seal [19,41]. As a result, it is comparatively very cheap and usable with cm scale microfluidic chips. Due to the simplicity of the methodology, Tesla valves were sealed by using a direct thermal bonding technique. Commercially available 22 × 22 mm polymer coverslips (Bel-Art) were used. The sealing process begins with a heating ablated sample on the heating plate to 180 ^∘^C degrees. The correct temperature was found experimentally. Too low temperature resulted in poor adhesion of polymer coverslip to the sample, while too high temperature concluded in polymer flowing into the channel, fully clogging it. After the sample reached the correct temperature, a polymer coverslip was added and without any weight left to heat for 1 min and 30 s. The samples then were tested for any clogged parts by injecting red-colored water into the microfluidic device (Figure 8). No clogging or leaks were detected, proving it as an attractive candidate for sealing open laser-produced channel systems. Additionally, this method was used to seal 10 channel systems with no abnormalities or defects after the process. Therefore, it can also be considered highly repeatable.

### 3.3. Testing of the Tesla Valve

**Figure 9:**
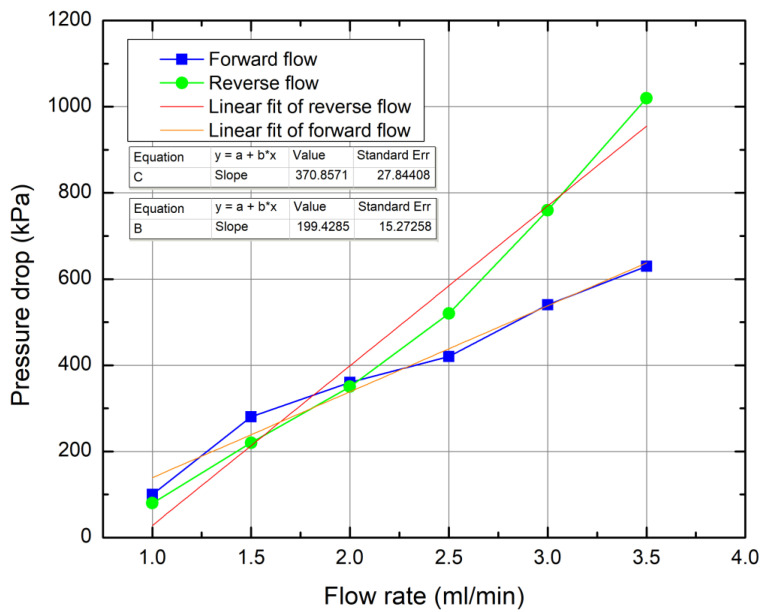
Pressure drop dependence on flow rate depicted graphically.

The testing experiments were conducted by injecting ethanol (ρ—789 kg/m^3^, µ—1.095 cP) into the microfluidic device. By changing the flow rates, different pressure drop readings were observed. The fluid was injected into the systems in two ways: forward and reverse flow. Observed pressure drops’ dependence on flow rate was depicted graphically (Figure 9). Since we are talking about microfluidic systems, it is important to evaluate the *R_E_* to determine if the flow is laminar or turbulent. The testing stage of the Tesla valve was done in a *R_E_* range from 76 to 281. As mentioned in the literature review, if the *R_E_* is below 2000, the microfluidic system falls upon the laminar flow regime.

From the observed data, it is clear that the manufactured Tesla valve works as intended—by injecting the fluid in reverse flow, higher pressure drop values are observed compared to injecting the fluid from a forward orientation. In addition, the graph was analyzed by linear fitting the data. From it, we can also see the difference in slope values (1.86 times difference), which indicates that the steeper line indicates pressure drop values occurring in reverse flow. Due to the Tesla valve’s unique geometry, with such an orientation, fluid is experiencing hydraulic resistance, while, in another direction, it is lower, i.e., the pressure drop values are lower as well. As the channels system presented in this work was based on theoretical work in [28], some comparisons can be made. First, it is important to note that the article in question dealt with a single Tesla valve segment, while the system produced in our work had eight segments. However, in both cases, a substantial difference can only be seen after the flow rate reaches >2 mL/min. In addition, if we consider that Tesla valve segments are stacked one after another in an additive fashion, the general pressure drop is very comparable. In our work, after eight segments, pressure drop at 3 ml/min in the reverse direction was ∼750 kPa, or 750/8 = 93.75 kPa for one segment. In [28], after one segment and γ = 2, pressure drop at 3 mL/min in the reverse direction was ∼80 kPa. This is remarkably close showing that produced Tesla valve operates very close to theory. This once again proves that there is negligible drag by channel walls, justifying the need to use optimized bi-burst fs fabrication.

What do presented results tell us about the feasibility of the usage of the Tesla valve in microfluidics? First off, the fact that the valve is operating shows that the bi-burst cut quality is sufficient and does not impede the functionality of the device. Furthermore, there is no substantial difference between forward and reverse flow at low pressures (bellow 600 kPa). This can be explained by the fact that the flow itself is impeding its movement in the channel due to the geometry of the valve. Thus, if the flow rate is low, the impeding effect is also low. On the contrary, when flow rates are above 600 kPa, the difference becomes rather substantial. This hints at the necessity to use Tesla valves only in applications where higher flow rates are employed. Here, please note that thermal-bonding-based sealing held presented pressure, showing it to be robust enough for this kind of application. In addition, the Tesla valve does not prevent general flow even at higher pressures. Therefore, it cannot be considered to be a perfect fluidic diode. Nevertheless, in some cases, just creating with a somewhat restricted flow can be enough. Then, the Tesla valve offers a very simple and cheap solution that does not require any additional steps for integration. This is in stark contrast to 3D micro-valves, which are produced additively and need to be integrated into channels during printing [42]. However, they offer the advantage of basically completely blocking reverse flow and can operate even at low pressures. Thus, when choosing a valve, both of these options are viable and should be considered depending on the circumstances.

## 4. Conclusions

In this work burst-mode, fs fabrication was optimized for glass microchannel fabrication. We found that only by optimizing *P* and *N* simultaneously can the best possible results be achieved. Namely, the surface roughness of the produced channel can be reduced from more than 1 µm down to 428 nm at *P*2*N*2. This does not impede manufacturing throughput, as the whole Tesla valve system with inlet and outlet can be made in around 15 min. Then, the channel was sealed using a simple thermal bonding technique. Despite the simplicity of such an approach, the seal held pressures exceeding 1 MPa during Tesla valve testing. Finally, due to good cut quality, the Tesla valve was shown to work well, when pressure is exceeding 600 kPa. This hints at its capabilities in fields where high pressure is used in microfluidic systems. However, it was also shown that it does not completely prevent reverse flow, which is the inherent property of all Tesla valves. Overall, acquired results also coincide well with previous theoretical works [28], proving that the micro Tesla valve is a viable design if flow rate regulation needs to be achieved without any moving parts.

## Figures and Tables

**Table 1:** Dimensions of the Tesla valve produced.

γ	*D*	*W*	*L*	α	β
2	150 µm	75 µm	400 µm	45^∘^	20^∘^

Where *γ*—channel width/depth aspect ratio, *D*—valve depth.

**Table 2:** Bi-burst mode processing for investigating surface roughness.

Conf.	*P*2*N*2	*P*4*N*4	*P*8*N*6	*P*12*N*8	*P*12*N*2	*P*16*N*4	*P*24*N*6
*R_A_* (nm)	428	730	926	1164	614	713	1219

## Data Availability

Not applicable.
